# Mechanisms of spinophilin-dependent pancreas dysregulation in obesity

**DOI:** 10.1152/ajpendo.00099.2023

**Published:** 2024-04-17

**Authors:** Kaitlyn C. Stickel, Nikhil R. Shah, Emily T. Claeboe, Kara S. Orr, Amber L. Mosley, Emma H. Doud, Teri L. Belecky-Adams, Anthony J. Baucum

**Affiliations:** ^1^Department of Biology, Indiana University-Indianapolis, Indianapolis, Indiana, United States; ^2^Medical Neurosciences and Medical Scientist Training Program, Indiana University School of Medicine, Indianapolis, Indiana, United States; ^3^Department of Pharmacology and Toxicology, Indiana University School of Medicine, Indianapolis, Indiana, United States; ^4^Center for Diabetes and Metabolic Diseases, Indiana University School of Medicine, Indianapolis, Indiana, United States; ^5^Department of Biochemistry and Molecular Biology, Indiana University School of Medicine, Indianapolis, Indiana, United States; ^6^Center for Proteome Analysis, Indiana University School of Medicine, Indianapolis, Indiana, United States; ^7^Center for Computational Biology and Bioinformatics, Indiana University School of Medicine, Indianapolis, Indiana, United States; ^8^Stark Neurosciences Research Institute, Indiana University School of Medicine, Indianapolis, Indiana, United States

**Keywords:** diabesity, protein phosphatase 1, obesity, scaffolding proteins, type 2 diabetes

## Abstract

Spinophilin is an F-actin binding and protein phosphatase 1 (PP1) targeting protein that acts as a scaffold of PP1 to its substrates. Spinophilin knockout (*Spino*^−/−^) mice have decreased fat mass, increased lean mass, and improved glucose tolerance, with no difference in feeding behaviors. Although spinophilin is enriched in neurons, its roles in nonneuronal tissues, such as β cells of the pancreatic islets, are unclear. We have corroborated and expanded upon previous studies to determine that *Spino*^−/−^ mice have decreased weight gain and improved glucose tolerance in two different models of obesity. We have identified multiple putative spinophilin-interacting proteins isolated from intact pancreas and observed increased interactions of spinophilin with exocrine, ribosomal, and cytoskeletal protein classes that normally act to mediate peptide hormone production, processing, and/or release in Lepr^db/db^ and/or high-fat diet-fed (HFF) models of obesity. In addition, we have found that spinophilin interacts with proteins from similar classes in isolated islets, suggesting a role for spinophilin in the pancreatic islet. Consistent with a pancreatic β cell type-specific role for spinophilin, using our recently described conditional spinophilin knockout mice, we found that loss of spinophilin specifically in pancreatic β cells improved glucose tolerance without impacting body weight in chow-fed mice. Our data further support the role of spinophilin in mediating pathophysiological changes in body weight and whole body metabolism associated with obesity. Our data provide the first evidence that pancreatic spinophilin protein interactions are modulated by obesity and that loss of spinophilin specifically in pancreatic β cells impacts whole body glucose tolerance.

**NEW & NOTEWORTHY** To our knowledge, these data are the first to demonstrate that obesity impacts spinophilin protein interactions in the pancreas and identify spinophilin specifically in pancreatic β cells as a modulator of whole body glucose tolerance.

## Graphical abstract

**Figure:**
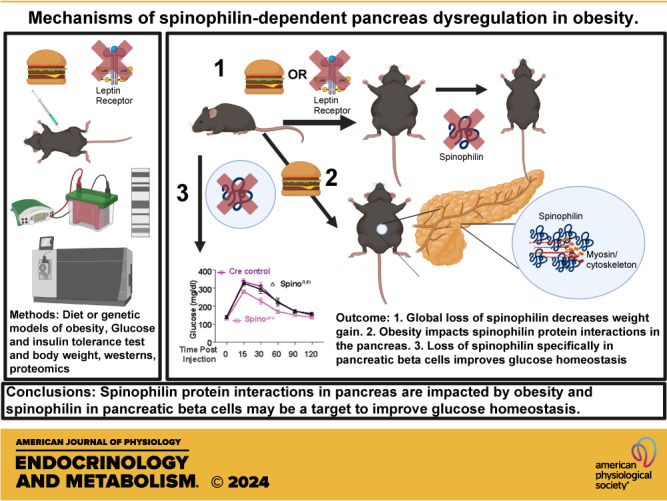

## INTRODUCTION

Chronic obesity is associated with pathophysiological changes that predispose individuals to dysregulation of glucose uptake and pancreatic β cell dysfunction that contribute to the development of type 2 diabetes (T2D) (1). The presentation of diabetes due to obesity, termed “diabesity,” is a major public health concern (2, 3). Obesity can lead to peripheral insulin resistance that, if not compensated for by increased insulin secretion by the β cells, will lead to dysregulated glucose uptake and hyperglycemia that defines T2D (4). To compensate for increases or decreases in peripheral (to the β cell) insulin sensitivity, the β cell can nonlinearly decrease or increase insulin secretion in response to glucose (4). Moreover, high glucose stimulation or cellular depolarization with potassium chloride induces biphasic insulin secretion. In isolated islets, the initial phase lasts ∼10 min and the more prolonged phase ∼25–35 min (5). The first phase is thought to involve readily releasable large dense core granules (LDCGs) that use multiple membrane receptors, including G-protein coupled receptors (GPCRs), ion channels, and receptor tyrosine kinases along with downstream kinase activation and calcium-dependent insulin exocytosis to respond to glucose stimulation (4). The second phase of insulin secretion is induced by movement of the reserve pool of LDCGs via cytoskeleton rearrangement (6). In addition to regulation of insulin by glucose-stimulated insulin secretion (GSIS), there is transcriptional, posttranscriptional, translational, and posttranslational regulation of insulin production and stability that can modulate the amount of insulin that is contained within LDCGs of the β cells (7–12). However, biochemical mechanisms that control processes such as β cell exhaustion that may link obesity and impaired pancreatic β cell function to diabetes, are poorly understood.

Spinophilin is a brain-enriched protein phosphatase 1 (PP1) targeting protein that is implicated in neuronal adaptations. Initial characterization of mice with global knockout (KO) of spinophilin (*Spino*^−/−^) found decreased body weight compared with wild-type (WT) mice (13). Moreover, more recent studies have found that *Spino*^−/−^ mice have improved glucose uptake and reduced weight gain, measures associated with improved insulin sensitivity and metabolic function (14, 15). Although we and others have characterized the importance of spinophilin in synaptic signaling mechanisms in the brain (13, 16–22), its expression and role in nonneuronal tissues such as the pancreas is less clear.

Previous studies identified that 16- to 18-wk-old chow-fed, male *Spino*^−/−^ mice had decreased fat mass, increased lean mass, and improved glucose tolerance (14). They proposed a β cell signaling mechanism from in vitro studies via M3 muscarinic acetylcholine receptors (M3R) for spinophilin’s involvement in negatively regulating M3R signaling and first-phase insulin release in MIN6 β cells (14). Moreover, recent studies found significant differences in weight gain, glucose uptake, and insulin sensitivity only in male spinophilin knockout (KO) versus WT mice on an 8-wk high-fat diet, with no significant differences in the female population, and proposed a mechanism involving spinophilin signaling in adipose tissue (15, 23). However, pancreas-specific mechanisms by which loss of spinophilin improves metabolic parameters are unknown.

In this study, we found that loss of spinophilin attenuates weight gain in both male and female Lepr^db/db^ and high-fat diet-fed (HFF) obese mice and improves glucose tolerance. Using unbiased proteomics approaches and targeted immunoblotting, we have found alterations in spinophilin interactions in the pancreas isolated from different obesity mouse models. Specifically, we identified overall increases in spinophilin protein interactions in the pancreas of HFF mice with proteins that are classically involved in signaling, protein translation, and cytoskeletal rearrangement, pathways that are all critical in hormone processing/release. Although it is unclear if these changes are occurring in exocrine or endocrine tissue, we found that loss of spinophilin specifically in insulin-producing β cells improves glucose tolerance in a cohort of young, chow-fed, male mice. Overall, we found that while spinophilin decreases weight gain, it improves glucose tolerance via pancreatic β cell-specific mechanisms, potentially via its interactions with multiple proteins involved in hormone production, processing, and release. These data position spinophilin at multiple points within the pancreas and pancreatic β cells to regulate diabesity.

## MATERIALS AND METHODS

### Animals

All animal studies were performed in accordance with the Guide for the Care and Use of Laboratory Animals and approved by the School of Science Institutional Animal Care and Use Committee (SC270R, SC310R) at Indiana University-Purdue University, Indianapolis (IUPUI). Male and Female *Lepr*^db/+^ mice [B6.BKS(D)-Leprdb/J, Stock No. 000697, Jackson Laboratories, Bar Harbor, ME] and whole body heterozygous spinophilin mice (Stock No. 018609; RRID: MMRRC_049172-UCD) were initially purchased from Jackson Laboratories and breeding colonies were maintained at IUPUI. Mice containing loxP sites around exon 3 of the *Ppp1r9b* (spinophilin) gene (Spino^fl/fl^) were generated by the University of Michigan and recently described (24). Ins1Cre mice [B6(Cg)-Ins1tm1.1(cre)Thor/J; Stock No. 026801] (25) and *Ins2*^Akita^ (C57Bl/6-Ins2^Akita^/J; Stock No. 003548) (26) mice were from Jackson laboratories. All animals were provided with chow and water ad libitum and group-housed. Mice were maintained on a 12-h light/dark schedule (7:00 am–7:00 pm–7:00 am). Leptin receptor heterozygous mutant mice (*Lepr*^db/+^) were crossed with heterozygous spinophilin mice (*Spino*^+/−^) to generate *Lepr*^db/+^/*Spino*^+/−^ male and female breeders. These mice were crossed to generate the following genotypes that were used for the genetic obesity model studies: *Lepr*^+/+^/*Spino*^+/+^, *Lepr*^db/db^/*Spino*^+/+^, *Lepr*^+/+^/*Spino*^−/−^, and *Lepr*^db/db^/*Spino*^−/−^. Mice were weighed bi-weekly from 4 wk until 20 wk of age. These mice were provided with standard chow (LabDiet, St. Louis, MO, Diet No. 5001, 23% protein, 4.5% fat, 6% fiber) and water ad libitum. For HFF mice, spinophilin WT and *spinophilin*^−/−^ male and female mice were weaned at *postnatal day* (*P*) *21* and placed on a high-fat diet (Research Diets, Inc., New Brunswick, NJ, D12492, 60% fat) at *P28*. These mice were weighed bi-weekly until 20 wk of age. Spinophilin floxed mice were crossed with Ins1Cre mice to generate Spino^ΔIns^ line. These mice were placed on standard chow ad libitum after weaning at *P21* and weighed bi-weekly until 18 wk of age. These mice were sacked via decapitation without anesthesia at 20 wk of age and pancreas was dissected, frozen in liquid nitrogen, and stored at −80°C for further biochemical studies. No a priori sample size calculation was performed. Not all mice were measured every other week for weights, so the total number of animals per group was different at different weeks; however, an *n* of at least 4 per time point was used for all weights. For area under the curve, weights at 6, 8, and 10 wk, and immunoblotting quantitation, each individual data point is shown.

### Intraperitoneal Glucose Tolerance Test

 Fasting glucose tolerance tests (GTTs) were performed at 6 and 10 wk of age. Mice were fasted for 4 h and an initial blood glucose reading was taken. Mice were injected intraperitoneally with glucose (2 g dextrose/kg body wt) and blood glucose was measured 15-, 30-, 60-, 90-, and 120-min postinjection using blood glucose test strips and monitor (Alpha-Trak2, Zoetis, Inc., Parsippany, NJ).

### Intraperitoneal Insulin Tolerance Test

Insulin tolerance tests (ITTs) were performed at 8 wk of age by fasting the mice for a short term (4 h). An initial blood glucose reading was taken before injection with 1 U/kg body wt of insulin (Humulin U-100, Eli Lilly and Co, Indianapolis, IN, Cat. No. 4273850). An intraperitoneal injection of insulin was given to the mice after the fasting period, and blood glucose was monitored at 15-, 30-, 60-, 90-, and 120-min postinjection using blood glucose test strips and monitor (Alpha-Trak2).

### Immunoprecipitation from WT, Ins2^Akita^, or Lepr^db/db^ Mice

Pancreatic tissue was dissected from WT, *Ins2*^Akita^, or *Lepr*^db/db^ mice. Tissue was homogenized in 2 mL of RIPA buffer (20 mM Tris HCl, 150 mM NaCl, 2 mM EDTA, 1× protease inhibitor cocktail (BiMake.com, Houston, TX), phosphatase inhibitors (20 mM sodium fluoride, 20 mM sodium orthovanadate, 20 mM β-glycerophosphate, and 10 mM sodium pyrophosphate; MilliporeSigma, St. Louis, MO or Thermo Fisher Scientific, Waltham, MA), 1% NP-40 (Thermo Fisher Scientific), and 1% deoxycholate (Thermo Fisher Scientific). Homogenates were sonicated and incubated with rotating for 1 h at 4°C. Homogenates were then centrifuged at 16,900 *g* for 10 min at 4°C. Goat anti-spinophilin antibody (10 µL) (Santa Cruz Biotechnology, Dallas, TX, Cat. No.14774—discontinued) or 3 µL of rabbit anti-spinophilin antibody (Cell Signaling Technologies, 9061S) were added to ∼400 µL of supernatants. Antibody was incubated for 1 h and then 20 µL of Protein G magnetic beads (Dynabeads, Life Technologies, Cat. No. 10009D) that had been washed three times in immunoprecipitate (IP) wash buffer [150 mM NaCl, 50 mM Tris-HCl pH 7.5, 0.5% (vol/vol) Triton X-100] were added. Beads were incubated for 1.5 h and then washed three times in IP wash buffer. Beads were eluted in 40 µL of 2× Laemmli sample buffer and 20 µL was run on a hand-cast SDS-PAGE gel for Coomassie staining and proteomics or immunoblotting.

#### Islet isolation.

Islets were isolated by the Center for Diabetes and Metabolic Diseases Islet and Physiology Core using previously described approaches (27).

### Coomassie Staining and Tryptic Digestion for Gel-C MS Proteomics

SDS-PAGE gels containing spinophilin immunoprecipitates were stained with an Imperial colloidal Coomassie stain (Thermo Fisher No. 24615) and regions of the gel were excised and tryptically digested as previously described (19, 21). For all steps, a sufficient volume of reagent was used that covered the gel pieces. Excised gels were destained [25 mM ammonium bicarbonate in 50% acetonitrile (ACN)]. DTT (10 mM) in 25 mM ammonium bicarbonate was added to reduce disulfides. Iodoacetamide (25 mM) was added to alkylate free-sulfhydryl groups and the reaction proceeded in the dark for 45 min. Gel pieces were subsequently incubated in 25 mM ammonium bicarbonate and then dehydrated with 25 mM ammonium bicarbonate in 50% ACN. The samples were then placed in a rotary vacuum and centrifuged until dry and subsequently digested with 12.5 ng/µL trypsin in 25 mM ammonium bicarbonate at 37°C overnight. Supernatants were collected from all samples. The remaining gel pieces were washed with 5% formic acid in 50% ACN and were vortexed and sonicated for 5 min.

### Immunoblotting

Immunoblotting was performed as previously described (19, 21). Briefly, SDS-PAGE gels were transferred using either a wet or semidry transblot turbo apparatus (Bio-Rad, Hercules, CA). Immunoblots were probed with goat anti-spinophilin antibody, mouse anti-myosin-9 antibody (MilliporeSigma MABT164), mouse anti-neurabin antibody (Santa Cruz Biotechnology, SC-136327), or mouse anti-PP1α antibody (Santa Cruz Biotechnology, SC-7482) and infrared secondary antibodies (From Jackson ImmunoResearch or Invitrogen) and developed on a Li-Cor Odyssey or Odyssey M (Li-Cor Biosciences, Lincoln, NE).

### Proteomics for Gel-C MS

Tryptic digestions from a colloidal Coomassie-stained gel underwent proteomics analysis on a Q-Exactive mass spectrometer using higher-energy collisional dissociation as previously described (21, 28). Specifically, digested samples were loaded onto a 100 µm × 2 cm Acclaim PepMap100 C18 nano trap column (5 µm, 100 Å) (Thermo Fisher) with an Ultimate 3000 liquid chromatograph (Thermo Fisher) at 5 µL/min. The peptides were separated on a silica capillary column that was custom-packed with C18 reverse phase material (Magic, 0.075 mm × 150 mm, 5 µm, 120 Å, Michrom Bioresources, Inc., Auburn, CA). The gradient was pumped at 300 nL/min from 10% to 45% solvent B (99.9% acetonitrile, 0.1% formic acid) for 87 min, then to 90% solvent B for 5 min, and reequilibrated to solvent A (99.9% water, 0.1% formic acid) for 12 min. The mass spectrometer was operated in a data-dependent acquisition mode controlled by the Xcalibur 2.2 software. Peptide mass spectra were acquired from an *m*/*z* range of 350–2,000 at a resolving power of 70,000 for 400 *m/z* ions. The top 15 most abundant multiply charged ions were subjected to higher-energy collisional dissociation (HCD) at a resolving power of 17,500 for 400 *m/z* ions. Ions with a charge state >+6 were rejected. Automatic gain control (AGC) targets were set to 3*e*6 for MS1 and 1*e*5 for data-dependent MS2 with an underfill ratio of 2.5%, given an intensity threshold of 5.0*e*4. A dynamic exclusion of 10.0 s was used.

Data were searched in Proteome Discoverer using a SEQUEST plug-in (v.1.4.1.14). The settings were: peptide tolerance of 10.0 ppm (monoisotopic), Fragment Tolerance of 0.020 Da (monoisotopic), variable modifications +16 on Met (oxidation), +42 on Lys (Acetylation), +57 on Cys (carbamidomethylation), +80 on Ser, Thr, Tyr (Phosphorylation), +80 on Ser, Thr (Sulfation). There were no fixed modifications. Tryptic database was searched and up to three missed cleavages were permitted. Data were loaded into Scaffold and then exported to Excel. Supplemental Tables are modified from the exported Excel table and the Scaffold file is included in the Supplemental Data (Scaffold_Supplement). All Supplemental Tables are available at https://doi.org/10.6084/m9.figshare.22507135.v1.

### Tandem Mass Tag and Qualitative Proteomics of Spinophilin Immunoprecipitates

Sample preparation, mass spectrometry analysis, bioinformatics, and data evaluation for quantitative proteomics and phosphoproteomics experiments were performed in collaboration with the Indiana University School of Medicine Center for Proteome Analysis similar to several previously published protocols (29, 30). Specifically, spinophilin was immunoprecipitated as described earlier and Protein G magnetic beads were washed three times in PBS. After washing, beads were covered with 8 M urea, 100 mM Tris hydrochloride, pH 8.5, reduced with 5 mM tris (2-carboxyethyl) phosphine hydrochloride (TCEP, Sigma Aldrich, Cat. No.: C4706) for 30 min at room temperature to reduce the disulfide bonds. The resulting free cysteine thiols were alkylated using 10 mM chloroacetamide (CAA, Sigma Aldrich, Cat. No.: C0267) for 30 min at RT, protected from light. Samples were diluted to 2 M urea with 50 mM Tris, pH 8.5, and proteolytic digestion was carried out with Trypsin/LysC Gold (0.3 µg, Mass Spectrometry grade, Promega Corporation, Cat. No.: V5072) overnight at 35°C. After digestion, samples were quenched with 0.4% trifluoroacetic acid (vol/vol, Fluka, Cat. No.: 91699), and the resultant peptides were desalted by solid-phase extraction using C18 Spin columns (Pierce, Cat. No.: 89870).

Peptides were reconstituted in 20 µL of 50 mM triethylammonium bicarbonate (TEAB, Sigma-Aldrich, Cat. No: T7408), pH 8.5, and labeled with 0.20 mg aliquots of TMT10plex Isobaric Label Reagent (Thermo Fisher Scientific, Cat. No.: 90111, Lot WG320953, Table 1). After 1 h incubation, the labeling reaction was quenched with 0.3% hydroxylamine (final vol/vol) for 15 min before combining the samples. The multiplexed sample was concentrated to dryness in a vacuum centrifuge, reconstituted with 0.1% Trifluoroacetic acid (TFA) aq. (vol/vol), desalted via Waters Sep-Pak Vac cartridge, and speed vacced to dryness. 1/10th of the total sample was then injected using an Easynano LC1200 coupled with 25 cm Aurora column (Ionopticks AUR2-25075C18A) on an Eclipse Orbitrap mass spectrometer (Thermo Fisher Scientific). Peptides were eluted over a 180-min method: Solvent B was increased from 5% to 30% over 160 min, to 85% B over 10 min, and down to 10% B (Solvent A: water, 0.1% formic acid; Solvent B: 100% acetonitrile, 0.1% formic acid). The mass spectrometer was operated in positive ion mode with 3 field symmetric ion mobility spectrometry compensation voltages (CVs) of −45, −55, −65. A cycle time of 1 s was used for each CV. MS1 parameters for each cycle were: orbitrap resolution of 120,000, scan range of 350–1,600 *m*/*z*, standard AGC, 50 ms max ion accumulation time (IT), minimum intensity of 2.5*e*4, precursor fit of 70% 0.7 *m*/*z*, charge state 2–6, 60 s dynamic exclusion. MS2 settings were quadrupole isolation of 0.7 *m*/*z*, fixed high-energy collisional dissociation (HCD) of 34, orbitrap resolution of 50,000, 200% AGC, dynamic max IT.

**Table 1.** Full pancreas proteomics

	Goat Spinophilin Antibody		Rabbit Spinophilin Antibody		
	WT	Lepr^db/db^	WT	Lepr^db/db^	Total Spectral Counts
Neurabin-2 (spinophilin)	21	17	42	36	116
Neurabin-1 (homolog-86%)	1	0	10	0	11
PP1α catalytic subunit	0	1	3	4	8
PP1ɣ catalytic subunit	0	1	3	5	9
BiP	2	25	10	34	71
Myosin-9	0	74	0	56	130

This table represents the top spinophilin interactors identified using MS-based analysis. This table shows spectral counts from goat and rabbit spinophilin antibodies individually, and total spectral counts are represented in the last column.

Data were analyzed in Proteome Discoverer 2.5. A *Mus musculus* protein database (UniProtKB/TrEMBL; last modified Jan 09, 2017) plus common laboratory contaminants was searched using SEQUEST HT. Precursor mass tolerance was set to 10 ppm and fragment mass tolerance set at 0.02 Da with a maximum of three missed cleavages. Dynamic modifications include methionine oxidation; deamidation of asparagine, phosphorylation on serine, threonine, and tyrosine, tandem mass tag (TMT) on lysine, acetyl on lysine, and GG + TMT (+343.206) on lysine residues. Dynamic peptide modifications were TMT at the N-terminus; dynamic protein terminus modifications were acetylation, met-loss, and met-loss plus acetylation. Static modifications were carbamidomethylation on cysteines. IMP-ptmRS node was used for localization scoring. Percolator false discovery rate (FDR) filtration of 1% was applied to both the peptide-spectrum match and protein levels. For the Proteome Discoverer consensus workflow, isobaric impurities corrections were turned on, the reporter ion coisolation threshold was set to 50%, and the average signal-to-noise threshold was 5. All peptides were used for protein roll-up, but modified peptides were excluded for pairwise ratio testing.

For analyses of WT/KO log2-fold change, one KO-HFD sample was excluded from the calculation due to a high abundance (TMT quantitation) of spinophilin in the sample, suggesting either carry-over or a mis-genotyping (e.g., heterozygous animal). In addition, one Chow-WT sample showed excessively high levels of cytoskeletal proteins, potentially suggesting nonspecific binding (e.g., levels 4–5 times higher than any other sample). Both the HFD-KO and the Chow-WT are shown in the Supplemental Table, but calculations for the tables in the text and for the stringdb were performed without these samples included. In addition, Supplemental Tables show all protein abundances detected in the mass spectrometry run. This includes contaminants that are listed below.

**Table:** 

Sample ID	TMT Label
WT_HFD	126
WT_HFD	127N
WT_HFD	128N
WT_StdChw	128C
WT_StdChw	129N
WT_StdChw	129C
spKO_HFD	130N
spKO_HFD	130C
spKO_HFD	131

For the qualitative proteomics of isolated islets, isolated islet samples were prepared as aforementioned through tryptic digesting and peptide cleanup. TMT labeling was not performed, each sample was injected individually on an EasyNano 1200 LC coupled to an Exploris 480 Orbitrap mass spectrometer with FAIMSpro installed (Thermo Fisher Scientific). Half of each sample was injected on a 25 cm EasySpray column (ES902) and run on a 90-min gradient. The mass spectrometer was operated in positive mode, APD on, default charge state of 2. Three FAIMS CVs were utilized (−40, −55, and −70) and each scan had identical parameters as follows: 1.3 s cycle time, MS1 resolution 120,000, MS1 scan range 375–1,500, MS1 AGC standard, MS1 max IT auto, charge states 2–7, intensity threshold of 5*e*3, and shared dynamic exclusion for 30 s, MS2 Isolation window of 1.6 *m*/*z*, HCD of 30, MS2 resolution of 15,000, first mass of 110 *m*/*z*, MS2 AGC standard and MS2 max IT of auto. Proteome Discoverer search settings were the same as earlier, other than no TMT modifications, and data were loaded into Scaffold 5 software (Proteome software) for spectral counting-based quantification. A list of all proteins along with spectral counts matching the mouse database is shown in the Supplemental Tables.

### Pancreas Fixation and β Cell Area Calculations

Fixation and β cell area calculations were performed by the Histology and Islet and Physiology Cores using previously described approaches (31). Additional details are in the Supplemental Information.

### Statistics

Analysis of curves was assessed by performing *t* tests, one-way ANOVAs, two-way ANOVAs, three-way ANOVAs, and appropriate post hoc tests. A Grubbs’s test was performed to identify outliers in data. Statistical significance was set at *P* value < 0.05. Specific statistical tests and subsequent post hoc tests with results are fully listed in the Supplemental Tables. All analyses were performed in Prism (GraphPad). For all studies, a single animal is the experimental unit.

## RESULTS

### Loss of Spinophilin Attenuates Weight Gain in Lepr^db/db^ Obese Mice and Mice on a HFF Diet

**Figure 1.**
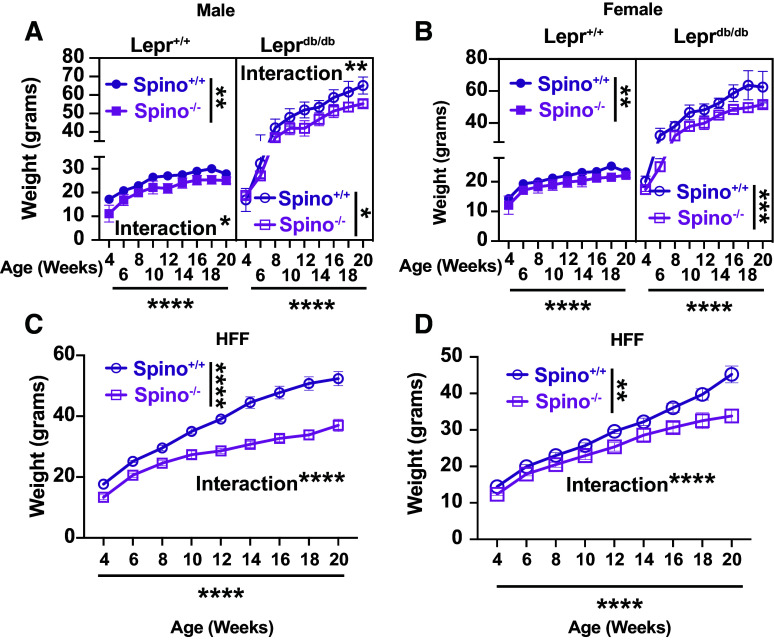
Loss of spinophilin significantly reduces weight gain in lean and two mouse models of obesity. *A* and *B*: male (*A*) or female (*B*) *Lepr*^+/+^/*Spino*^+/+^, Lepr^db/db^/*Spino*^+/+^, *Lepr*^+/+^/*Spino*^−/−^, and Lepr^db/db^/*Spino*^−/−^ mice weights were taken bi-weekly from 4 to 20 wk and plotted. A mixed-effects three-way ANOVA was performed initially, followed by a two-way ANOVA to determine the effect of spinophilin genotype, age, and an interaction within the Lepr^+/+^ and Lepr^db/db^ genotypes individually. *C* and *D*: male (*C*) and female (*D*) *Spino*^+/+^/high-fat diet-fed (HFF) and *Spino*^−/−^/HFF mice were weighed bi-weekly and plotted. For two-way ANOVAs, significant spinophilin genotype, time, and interaction (spinophilin genotype × time) are shown. *n* = 4–12 mice per group for each age point. Data ± SE are shown. *P < 0.05, **P < 0.01, ***P < 0.001, ****P < 0.001. All statistics are shown in Supplemental Material (https://doi.org/10.6084/m9.figshare.22507135.v1).

Previous studies established that loss of spinophilin resulted in reduced weight gain in HFF and chow-fed, male spinophilin KO compared with WT mice (14, 15) whereas HFF spinophilin KO females had no difference in body weight. Male and female *Spino*^−/−^/Lepr^db/db^ and *Spino*^−/−^/*Lepr*^+/+^ mice gained significantly less weight than their corresponding *Spino*^+/+^ littermates (Fig. 1, *A* and *B*). *Spino*^−/−^ male and female HFF mice also gained significantly less weight than *Spino*^+/+^ HFF littermates of the corresponding sex (Fig. 1, *C* and *D*). Therefore, loss of spinophilin in male and female mice significantly reduces weight gain in both lean mice and in multiple obesity models when measuring long-term weight changes and starting diet at a young age.

### Loss of Spinophilin Improves GTT in Obese (HFF) Male and Female Mice

**Figure 2.**
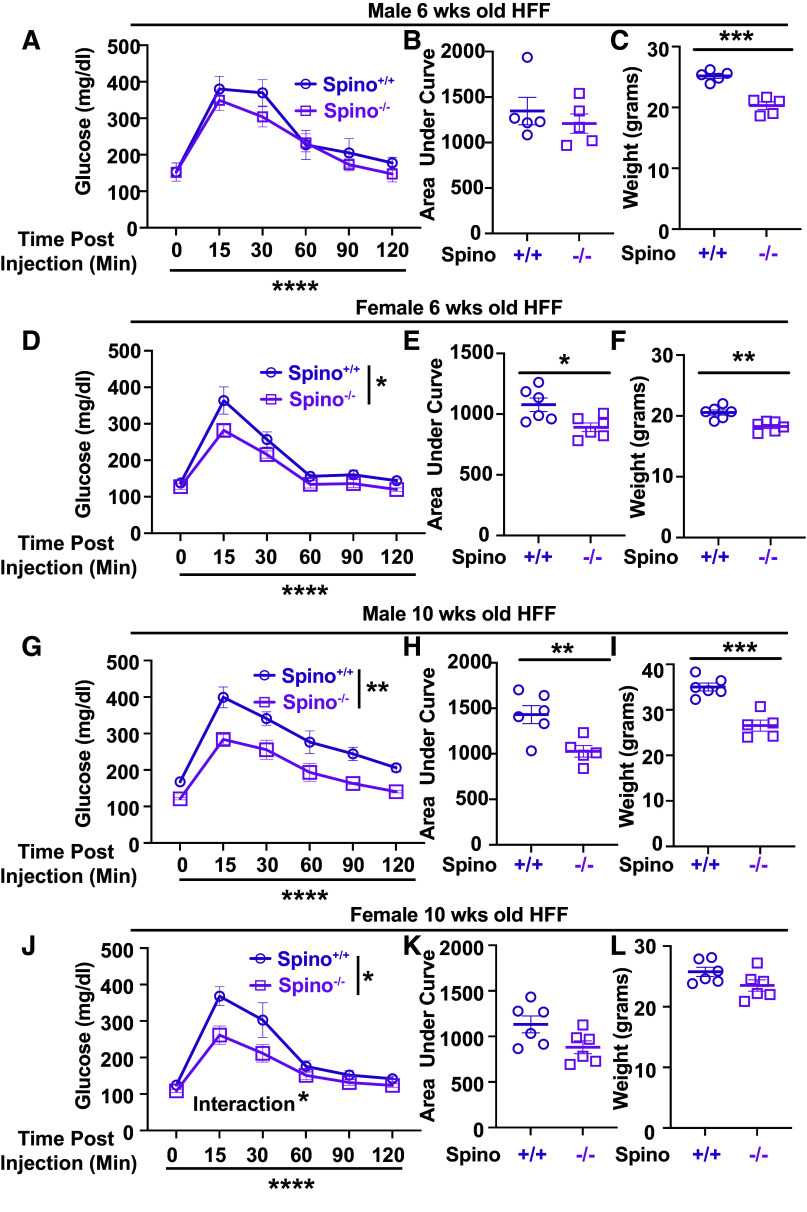
*Spino*^−/−^ mice have improved glucose tolerance test (GTT) in high-fat diet-fed (HFF) male and female mice. *A*: intraperitoneal glucose tolerance test (IPGTT) of HFF wild-type (WT) and spinophilin knockout (KO) male mice at 6 wk of age. *B*: area under the curve for the IPGTT from HFF WT and spinophilin KO male mice at 6 wk of age. *C*: weights from male HFF WT and spinophilin KO mice at 6 wk of age. *D*: IPGTT of HFF WT and spinophilin KO female mice at 6 wk of age. *E*: area under the curve for the IPGTT from HFF WT and spinophilin KO female mice at 6 wk of age. *F*: weights from female HFF WT and spinophilin KO mice at 6 wk of age. *G*: IPGTT of HFF WT and spinophilin KO male mice at 10 wk of age. *H*: area under the curve for the IPGTT from HFF WT and spinophilin KO male mice at 10 wk of age. *I*: weights from male HFF WT and spinophilin KO mice at 10 wk of age. *J*: IPGTT of HFF WT and spinophilin KO female mice at 10 wk of age. *K*: area under the curve for the IPGTT from HFF WT and spinophilin KO female mice at 10 wk of age. *L*: weights from female HFF WT and spinophilin KO mice at 10 wk of age. Data are given with SE. A two-way repeated-measures ANOVA (*A*, *D*, *G*, and *J*) or unpaired *t* tests (*B*, *C*, *E*, *F*, *H*, and *I*) were performed. For two-way ANOVA, significant spinophilin genotype, time, and interaction (spinophilin genotype × time) are shown. *n* = 5–6 mice per group. *P < 0.05, **P < 0.01, ***P < 0.001, ****P < 0.001. All statistics are shown in Supplemental Material (https://doi.org/10.6084/m9.figshare.22507135.v1).

Previous studies concluded that loss of spinophilin improves GTT in male 16- to 18-wk-old mice (14) and in 16-wk-old male mice on high-fat diets for 8 wk, with no significant difference in HFF female mice (15). Our HFF male and female *Spino*^−/−^ mice were placed on a high-fat diet starting at 4 wk of age and GTTs were performed at 6 and 10 wk of age. We found that loss of spinophilin in both male and female HFF mice had unique impacts on glucose tolerance at different ages. Specifically, male *Spino*^−/−^ HFF mice had no significant difference in glucose tolerance at 6 wk of age (Fig. 2, *A* and *B*) but weighed significantly less than WT mice (Fig. 2*C*). At 10 wk of age, male *Spino*^−/−^ mice had both significantly decreased GTT and significantly decreased body weights (Fig. 2, *D–F*). At 6 wk of age, female *Spino*−/− mice had decreased GTT (Fig. 2, *G* and *H*) and body weight (Fig. 2*I*). At 10 wk of age, there was a significant genotype effect on the GTT and a time × genotype interaction. However, there was only a trend for a decreased area under the curve for the GTT (Fig. 2, *I* and *J*) and no significant difference in body weight between the two groups (Fig. 2*K*).

### Obesity Modulates Spinophilin Interactions in the Pancreas

**Figure 3.**
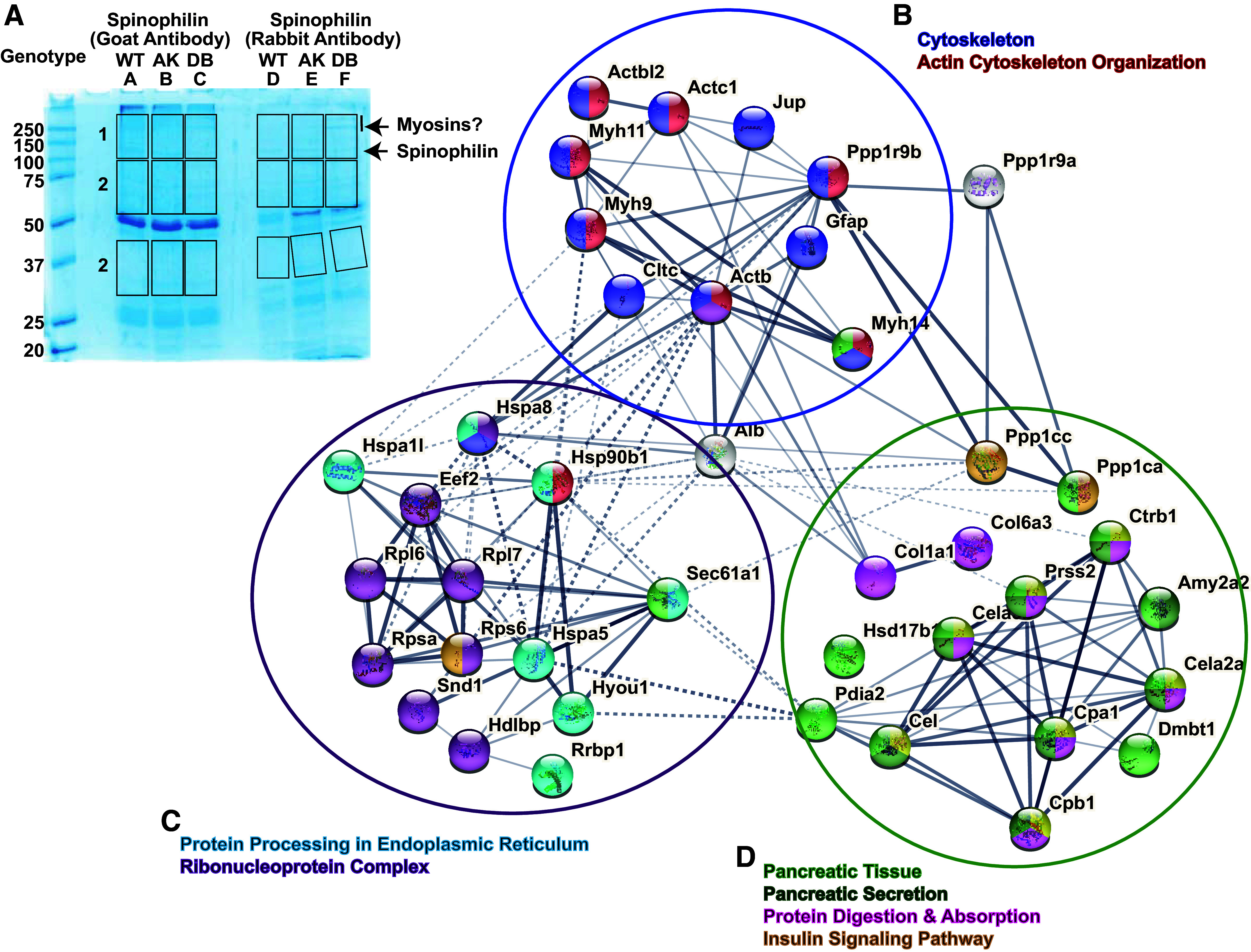
Obesity-induced changes in the spinophilin protein interactome. Spinophilin was immunoprecipitated from pancreas of a single 6-wk-old wild-type (WT) and Lepr^db/db^ mice using two different spinophilin antibodies. Immunoprecipitates were separated by SDS-PAGE, stained (*A*), and excised for MS-based analysis. Sixty-six total interacting proteins were detected with two or more spectral counts (Supplemental Table S3) but for more targeted expression, we only considered proteins with spectral counts of four or above (Supplemental Table S2). *B*–*D*: representation of Kyoto Encyclopedia of Genes and Genomes (KEGG) pathway analysis from STRING input. Spinophilin interactors altered in obesity have been sorted into three clusters based on common biological processes. Interactors with a spectral count of four or more were included. *B*: spinophilin interactors clustered into common cytoskeletal functions. *C*: spinophilin interactors clustered into common biological functions involved in protein processing in the endoplasmic reticulum and ribonucleoprotein complexes. *D*: spinophilin interactors clustered into common pancreatic functions, such as secretion, digestion, and insulin signaling.

The weight gain and GTT data described earlier suggest that loss of spinophilin may impact obesity and glucose tolerance independently. Although previous studies using an immortalized mouse insulinoma β cell line (MIN-6) demonstrated a spinophilin-dependent regulation of M3 muscarinic receptor-dependent insulin secretion (14), the role of spinophilin in vivo in the intact pancreas has not been probed. Using proteomics and immunoblotting-based approaches (Fig. 3*A*), we identified multiple putative spinophilin-interacting proteins from whole pancreas lysates isolated from WT and *Lepr*^db/db^ mice by immunoprecipitating for spinophilin and subjecting immunoprecipitates to in-gel tryptic digestion followed by mass spectrometry (Fig. 3, *B–D*, Table 1; Supplemental Tables S2 and S3). We used STRING database (stringdb) (32) as part of the ELIXIR infrastructure to perform the Kyoto Encyclopedia of Genes and Genomes (KEGG) and Gene Ontology (GO) pathway analyses (Supplemental Tables S4, S5, S6, and S7) on all spinophilin-interacting proteins identified in our proteomics experiment with a total spectral count of 4 or greater. We then clustered similar proteins together based on specific biological processes, cellular component/localization, and KEGG pathways (Fig. 3, *B–D*). Spinophilin contains an F-actin binding domain and is important in cytoskeletal rearrangement in dendritic spines (16, 33). Here, we also identified that spinophilin interacts with different classes of myosins and actins in the pancreas that are important in cytoskeletal organization (34–36) (Fig. 3*B*). Myosin-9 was identified to have the greatest difference between the number of spectral counts observed in the WT and *Lepr*^db/db^ mice. Interestingly, we observed multiple spinophilin-interacting proteins involved in protein translation in the pancreas, including ribosomal proteins, heat shock proteins, and endoplasmic reticulum (ER)-chaperones that are upregulated in ER stress conditions such as BiP and protein disulfide isomerase (PDI) (Fig. 3*C*). Moreover, we concluded that spinophilin interacts with proteins classically identified in pancreatic secretion, insulin signaling, and protein digestion (Fig. 3*D*).

### Spinophilin-Interacting Proteins in Different Models of Diabetes

**Figure 4.**
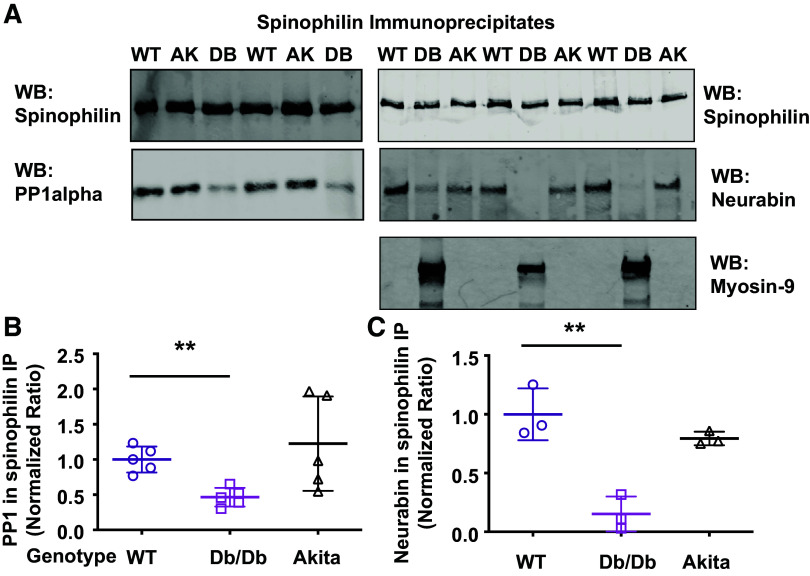
Spinophilin protein interactions with protein phosphatase 1 (PP1), neurabin, and myosin-9 in wild-type (WT), Ins2^Akita^, and Lepr^db/db^ mice. *A*: spinophilin was immunoprecipitated from pancreas of adult WT, Ins2^Akita^ (AK), and Lepr^db/db^ (DB) mice. Immunoblotting for spinophilin, PP1α, myosin-9, and neurabin was performed. *B* and *C*: PP1α (*B*) or neurabin (*C*) expression in spinophilin immunoprecipitates was normalized to spinophilin expression in the immuno precipitate. A normalized ratio was plotted. *n* = 3–5 mice per group. **P < 0.01. All statistics are shown in Supplemental Material (https://doi.org/10.6084/m9.figshare.22507135.v1).

We immunoprecipitated spinophilin from pancreatic lysates and immunoblotted for interacting proteins observed in our initial proteomics study, including neurabin, PP1α, and myosin-9 in control mice and two different models of glucose intolerance, *Ins2*^Akita^ mice, which develop insulin resistance but not obesity, and *Lepr*^db/db^ mice that are both obese and insulin resistant. It is important to note that many of the proteins that were detected by proteomics in the spinophilin immunoprecipitates were not detected in pancreatic lysate Western blots, in part, due to the low abundance of these proteins. However, spinophilin and associated proteins were readily detected when enriched by spinophilin immunoprecipitation. We observed a decreased PP1 and spinophilin association in the type 2 diabetic mouse model (Lepr^db/db^), but not in the Ins2^Akita^ mice, compared with WT mice. We observed a significant decrease in the association of spinophilin with its homolog neurabin in *Lepr*^db/db^, but not *Ins2*^Akita^ mice, compared with WT mice. Moreover, we only observed a Western blot band with spinophilin and myosin-9 coimmunoprecipitation in our *Lepr*^db/db^ mice, with no association in WT or *Ins2*^Akita^ pancreas (Fig. 4). This further confirmed the obesity-dependent increase in myosin-9 spectral counts in spinophilin immunoprecipitates observed in our proteomics study (Table 1).

### TMT Proteomics of HFF WT and Spinophilin KO Mouse Pancreas

To validate and quantify these interactions in a nongenetic, and more human-relevant, model of obesity and to determine the specificity of the interactions with spinophilin, we immunoprecipitated spinophilin from the pancreas of WT lean male mice on standard chow and WT HFF obese male mice. In addition, we used HFF spinophilin KO mice as a critical negative interaction control (18). Immunoprecipitates were analyzed using a ratiometrically quantitative tandem mass tag (TMT) proteomics experiment. To be considered for our specificity cutoff and to remove any contaminates or nonspecific interactors, we filtered our samples with a WT/KO log_2_ fold-enrichment of 0.5 and then removed any protein with less than two unique peptides. We then normalized the abundance of these specific coprecipitating proteins to the abundance of spinophilin in the corresponding sample (Supplemental Tables). All proteins detected regardless of specificity are shown in the Supplemental Tables.

**Figure 5.**
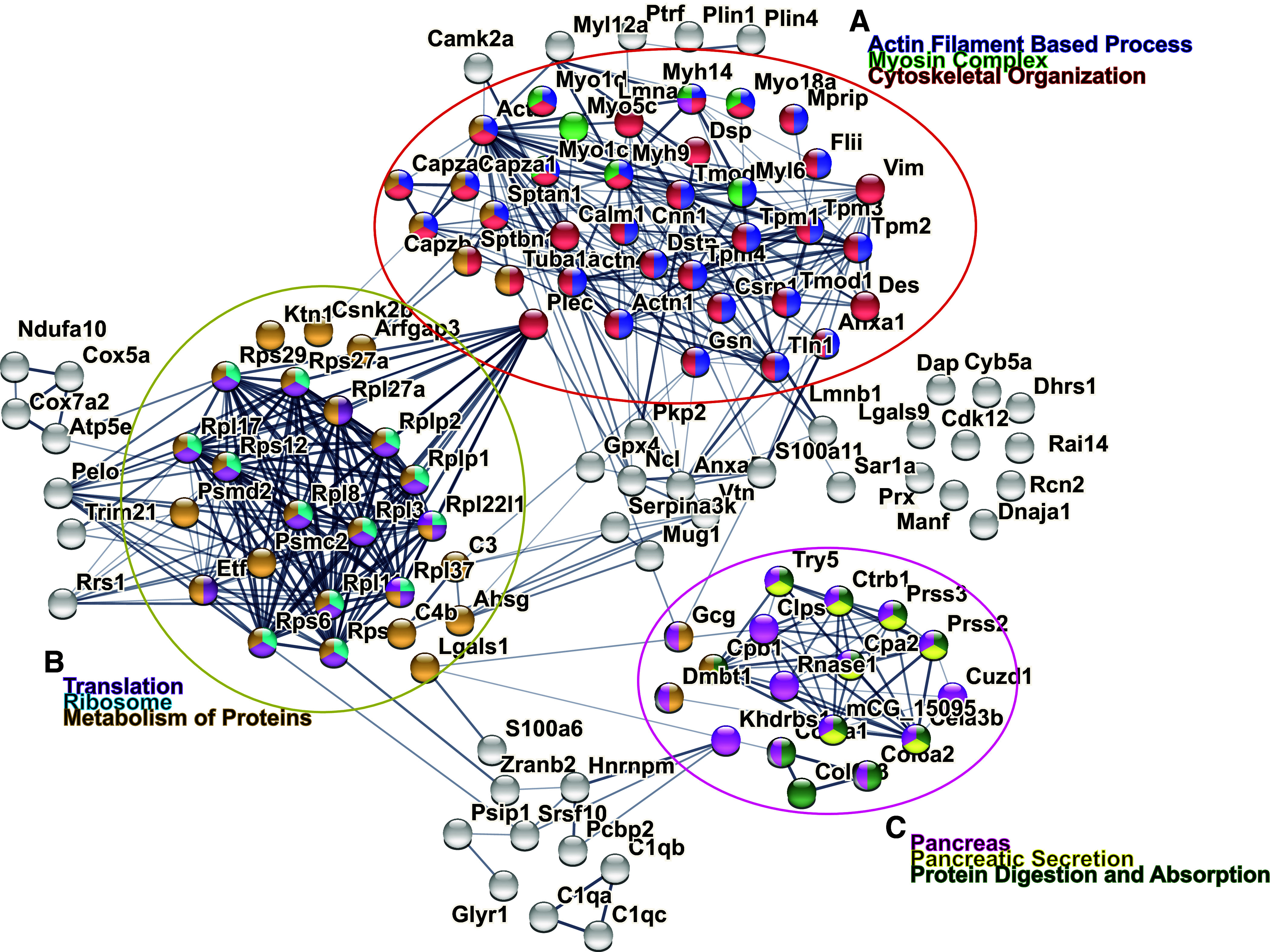
Visual representation of Kyoto Encyclopedia of Genes and Genomes (KEGG) and Gene Ontology (GO) pathway analysis from STRING-db input of spinophilin immunoprecipitates from pancreas isolated from lean and high-fat diet-fed (HFF) mice. Spinophilin was immunoprecipitated from the pancreas of three wild-type (WT) lean male mice on standard chow, three WT HFF obese male mice, and three HFF *Spino*^−/−^ mice as a control. Immunoprecipitates were subjected to a tandem mass tag proteomics experiment. We then filtered our samples with a WT/knockout (KO) log_2_ fold-enrichment of ≥0.5, removed any protein with less than two unique peptides, and normalized to the spinophilin abundance in the corresponding sample. *A*: top spinophilin interactors are considered to have an obesity-dependent increase (log_2_-fold change) clustered together based on common functions in cytoskeletal organization. *B*: top spinophilin interactors increased in obesity clustered into common biological functions, such as translation, metabolism of proteins, and ribosomes. *C*: top spinophilin interactors increased in obesity clustered into common biological expression and function, including pancreas, pancreatic secretion, and digestion. A list of all spinophilin interactors in these clusters is provided in Table 2.

Overall, we replicated specific increased interactions with myosin-9 (Table 2) and BiP but did not observe a quantitative change in PP1 interaction with spinophilin in this obesity model. However, BiP protein was detected equally in WT and spinophilin KO HFF mice, suggesting an obesity-induced increase in this protein, but a nonspecific pulldown (Supplemental Tables). We performed STRING, KEGG, and GO pathway analyses of specific spinophilin-interacting proteins that also had an increased interaction (log_2_-fold change of ≥0.5in HFF vs. lean) (Supplemental Tables). We observed multiple proteins important in cytoskeletal organization (Fig. 5*A*), translation (Fig. 5*B*), and pancreatic secretion (Fig. 5*C*).

**Table 2.** Spinophilin interactors-clusters

	Gene	Log2 Normalized Ratio	No. PSMs
Exocrine and Digestion			
Ribonuclease pancreatic	*Rnase1*	1.73	11
MCG15083	*TRY5*	1.61	26
Carboxypeptidase B1	*Cpb1*	1.58	48
Anionic trypsin-2	*Prss2*	1.45	12
Carboxypeptidase A2	*Cpa2*	1.42	11
Chymotrypsin-like elastase family member 3B	*Cela3b*	1.42	24
Colipase	*Clps*	1.39	17
Chymotrypsinogen B	*Ctrb1*	1.35	51
Protein Prss3	*Prss3*	1.34	17
CUB and zona pellucida-like domain-containing protein 1	*Cuzd1*	0.93	12
Deleted in malignant brain tumors 1 protein	*Dmbt1*	0.77	112
KH domain-containing, RNA-binding, signal transduction-associated protein 1	*Khdrbs1*	0.79	2
Collagen α-2 (VI) chain	*Col6a2*	1.49	6
Collagen α-1 (VI) chain	*Col6a1*	1.31	7
Protein Col6a3	*Col6a3*	0.83	19
Endocrine Pancreas			
Glucagon	*Gcg*	1.58	5
Ribosomal/Translation			
Perilipin-4	*Plin4*	1.97	42
60S acidic ribosomal protein P2	*Rplp2*	1.46	21
Eukaryotic peptide chain release factor subunit 1	*Etf1*	1.4	3
α-2-HS-glycoprotein	*Ahsg*	1.32	3
Perilipin-1	*Plin1*	1.3	25
Galectin-1	*Lgals1*	1.26	11
Kinectin	*Ktn1*	1.01	2
Casein kinase II subunit β	*Csnk2b*	0.96	2
40S ribosomal protein S12	*Rsp12*	0.94	17
60S ribosomal protein L37	*Rpl37*	0.94	15
ADP-ribosylation factor GTPase-activating protein 3	*Arfgap3*	0.93	9
40S ribosomal protein S24	*Rps24*	0.84	49
60S ribosomal protein L11	*Rpl11*	0.81	150
Ribosome biogenesis regulatory protein homolog	*Rrs1*	0.8	3
60S ribosomal protein L17	*Rpl17*	0.71	109
60S ribosomal protein L27a	*Rpl27a*	0.68	52
60S ribosomal protein L8	*Rpl8*	0.64	222
40S ribosomal protein S29	*Rps29*	0.62	8
60S ribosomal protein L22-like 1	*Rpl22l1*	0.61	17
60S ribosomal protein L3	*Rpl37*	0.61	219
40S ribosomal protein S6	*Rps6*	0.56	124
Ubiquitin-40S ribosomal protein S27a	*Rps27a*	0.53	28
Cytoskeleton			
Talin-1	*Tln1*	1.93	7
Annexin A1	*Anxa1*	1.6	2
Protein Myl12a	*Myl12a*	1.52	31
Unconventional myosin-lc	*myo1c*	1.21	20
α-Actinin-4	*Actn4*	1.06	23
Destrin	*Dstn*	1.05	11
Spectrin β chain, nonerythrocytic 1	*Sptbn1*	1.03	22
Plectin	*Plec*	1.01	151
MCG5400	*Myl12a*	1.01	95
Gelsolin	*Gsn*	0.98	35
Myosin phosphatase Rho-interacting protein	*Mprip*	0.97	27
Desmoplakin	*Dsp*	0.96	67
Spectrin α chain, nonerythrocytic 1	*Sptan1*	0.96	36
Myosin-14	*Myh14*	0.94	170
Tropomyosin α-4 chain	*Tpm4*	0.94	25
Actin, cytoplasmic 1	*Actb*	0.93	279
Capping protein (Actin filament) muscle Z-line, β, isoform CRA_a	*Capzb*	0.92	28
F-actin-capping protein subunit α-1	*Capza1*	0.91	11
Tubulin α-1A chain	*Tuba1a*	0.91	53
Tropomyosin α-3 chain	*Tpm3*	0.9	56
Tropomodulin-3	*Tmod3*	0.87	55
Myosin-9	*Myh9*	0.86	1393
Cysteine and glycine-rich protein 1	*Csrp1*	0.82	3
Protein Myo5c	*Myo5c*	0.81	76
Desmin	*Des*	0.79	72
F-actin-capping protein subunit α-2	*Capza2*	0.75	18
Protein flightless-1 homolog	*Flii*	0.72	6
Unconventional myosin-XVIIIa	*Myo18a*	0.71	31
Myosin light polypeptide 6	*Myl6*	0.7	94
Tropomodulin-1	*Tmod1*	0.7	11
Myosin phosphatase Rho-interacting protein	*Mprip*	0.7	26
α-Actinin 1a	*Actn1*	0.69	25
Calponin-1	*Cnn1*	0.63	2
Tropomyosin α-1 chain	*Tpm1*	0.6	93
Unconventional myosin-Id	*Myo1d*	0.55	29
Tropomyosin β chain	*Tpm2*	0.52	62

### Spinophilin Interactome in Islets

**Figure 6.**
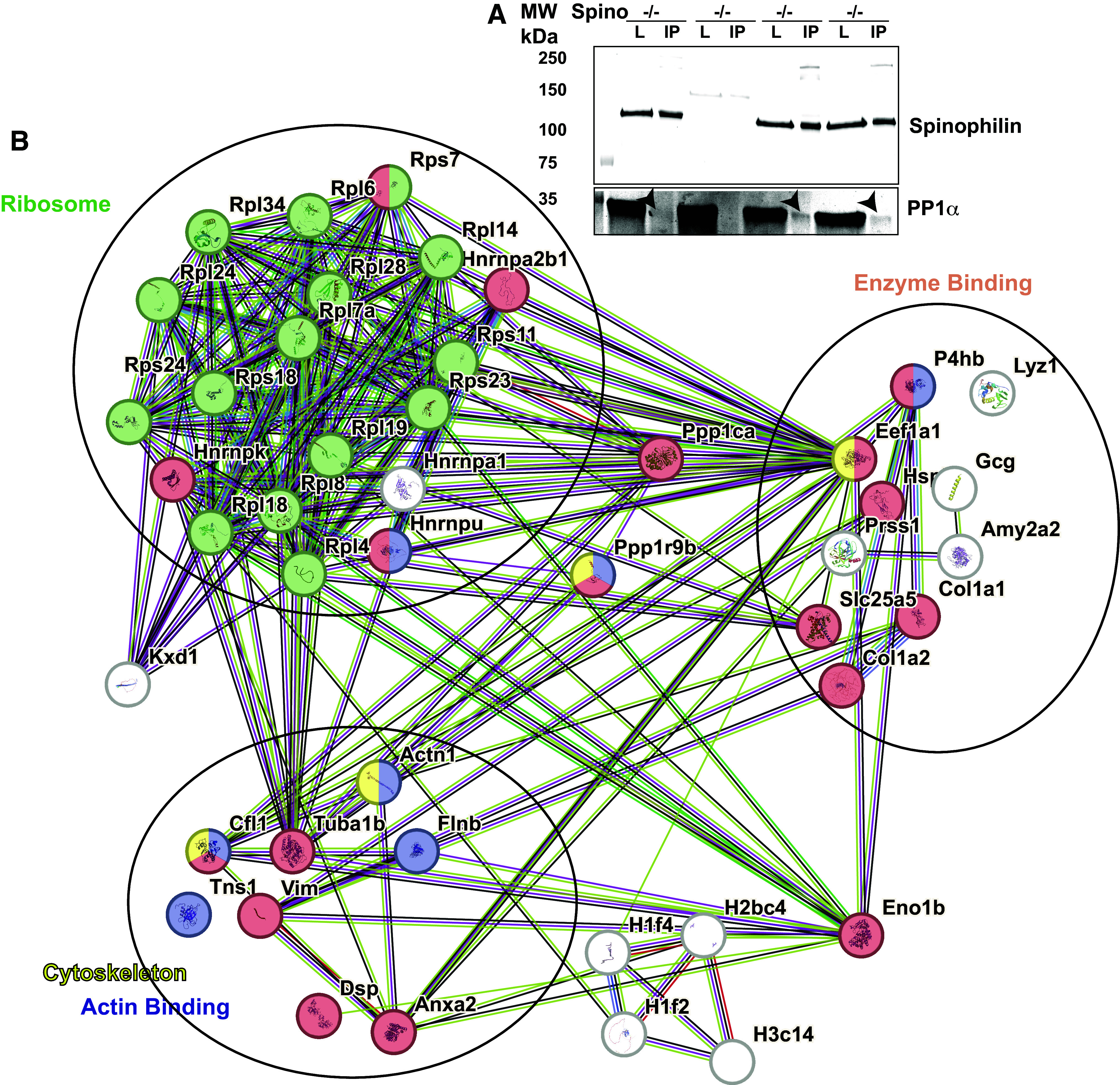
Visual representation of Gene Ontology (GO) pathway analysis from STRING-db input of spinophilin immunoprecipitates from isolated islets. Spinophilin was immunoprecipitated from islets isolated from three wild-type (WT) and one *Spino*^−/−^ mice. Immunoprecipitates were subjected to mass spectrometry and database search. We then filtered our samples to eliminate keratins, immunoglobulins, contaminants, and nonmouse proteins. Only those interactions that were specifically detected in at least one of the three WT samples and not present in the knockout (KO) or those peptides detected at an average of two times greater than the KO sample were input into the STRING-db program. *A*: Western blot of spinophilin and PP1α in lysates (L) or spinophilin immunoprecipitates (IP) from WT and *Spino*^−/−^ mice (KO). *B*: String-db output clustered into ribosome, enzyme binding, and cytoskeletal proteins.

To begin to address the role of spinophilin specifically in islets, we immunoprecipitated spinophilin from isolated islets from *Spino*^+/+^ or *Spino*^−/−^ mice and performed qualitative proteomics analysis of spinophilin immunoprecipitates from the isolated islets. Western blotting of isolated islets showed spinophilin in both lysates from *Spino*^+/+^, but not *Spino*^−/−^, mice (Fig. 6*A*). Moreover, PP1α co-immunoprecipitated with spinophilin in *Spino*^+/+^, but not *Spino*^−/−^, islets; however, the coimmunoprecipitation was very low (Fig. 6*A*). In addition to immunoblotting, we detected an average of 6.3 spectral counts per islet sample isolated from *Spino*^+/+^ mice and 0 spectral counts matching spinophilin in the 1 *Spino*^−/−^ mouse (Table 3). In addition, specific interacting proteins are shown in Table 3. A full list of all proteins identified is provided in the Supplemental Tables. Spinophilin-interacting proteins were input into the String-DB program and show overlapping interactions with proteins and or/proteins from similar classes as those detected in total pancreas immunoprecipitates (Fig. 6*B*). The full gene ontology pathways matching the detected proteins are given in the Supplemental Tables.

**Table 3.** Spinophilin-interacting proteins detected in isolated islets

		Spectral Counts					
Gene Name	Molecular Weight	KO	WT	WT	WT	Total Counts in WT	Fold-Enrichment
*Ppp1r9b*	90 kDa	0	8	6	5	19	#DIV/0!
*H1-4*	22 kDa	0	3	6	6	15	#DIV/0!
*LOC665622*	15 kDa	0	2	3	1	6	#DIV/0!
*Tuba1b*	50 kDa	0	0	3	3	6	#DIV/0!
*Dsp*	333 kDa	0	1	2	0	3	#DIV/0!
*Amy2*	57 kDa	0	1	2	0	3	#DIV/0!
*H3c14*	20 kDa	0	0	2	0	2	#DIV/0!
*Rps24*	14 kDa	0	0	1	1	2	#DIV/0!
*Rpl8*	28 kDa	0	0	1	1	2	#DIV/0!
*Rpl7a*	30 kDa	0	0	1	1	2	#DIV/0!
*Rpl18*	19 kDa	0	0	1	1	2	#DIV/0!
*Hnrnpa1*	34 kDa	0	0	1	1	2	#DIV/0!
*Col1a2*	130 kDa	0	0	2	0	2	#DIV/0!
*P4hb*	57 kDa	0	0	1	1	2	#DIV/0!
*Rpl14*	24 kDa	0	0	1	1	2	#DIV/0!
*Ppp1ca*	38 kDa	0	1	0	0	1	#DIV/0!
*Hnrnpu*	88 kDa	0	0	0	1	1	#DIV/0!
*Slc25a5*	33 kDa	0	0	0	1	1	#DIV/0!
*Rpl28*	16 kDa	0	0	1	0	1	#DIV/0!
*Rpl24*	18 kDa	0	0	1	0	1	#DIV/0!
*Rpl34*	13 kDa	0	0	0	1	1	#DIV/0!
*Tns1*	183 kDa	0	0	1	0	1	#DIV/0!
*Hnrnpa2b1*	37 kDa	0	0	0	1	1	#DIV/0!
*Rps18*	18 kDa	0	0	0	1	1	#DIV/0!
*Rpl6*	34 kDa	0	0	1	0	1	#DIV/0!
*Col1a1*	138 kDa	0	0	1	0	1	#DIV/0!
*Kxd1*	22 kDa	0	0	1	0	1	#DIV/0!
*Actn1*	103 kDa	0	0	1	0	1	#DIV/0!
*Flnb*	278 kDa	0	0	0	1	1	#DIV/0!
*EG433182*	47 kDa	0	0	1	0	1	#DIV/0!
*Rpl4*	37 kDa	0	0	0	1	1	#DIV/0!
*Cfl1*	19 kDa	0	0	0	1	1	#DIV/0!
*Rps11*	19 kDa	0	0	1	0	1	#DIV/0!
*Rps23*	16 kDa	0	0	1	0	1	#DIV/0!
*Gcg*	21 kDa	0	0	0	1	1	#DIV/0!
*Rpl19*	23 kDa	0	1	0	0	1	#DIV/0!
*Rps7*	22 kDa	0	0	1	0	1	#DIV/0!
*Hnrnpk*	50 kDa	0	0	1	0	1	#DIV/0!
*Anxa2*	39 kDa	0	0	0	1	1	#DIV/0!
*Hspb1*	9 kDa	0	0	0	1	1	#DIV/0!
*Prss1*	26 kDa	0	0	0	1	1	#DIV/0!
*Lyz1*	17 kDa	0	0	0	1	1	#DIV/0!
*H1-2*	21 kDa	1	3	4	5	12	4
*Vim*	54 kDa	1	1	4	5	10	3.33333333
*Eef1a1*	50 kDa	1	1	3	2	6	2

### Pancreatic β Cell-Specific Spinophilin Knockout

**Figure 7.**
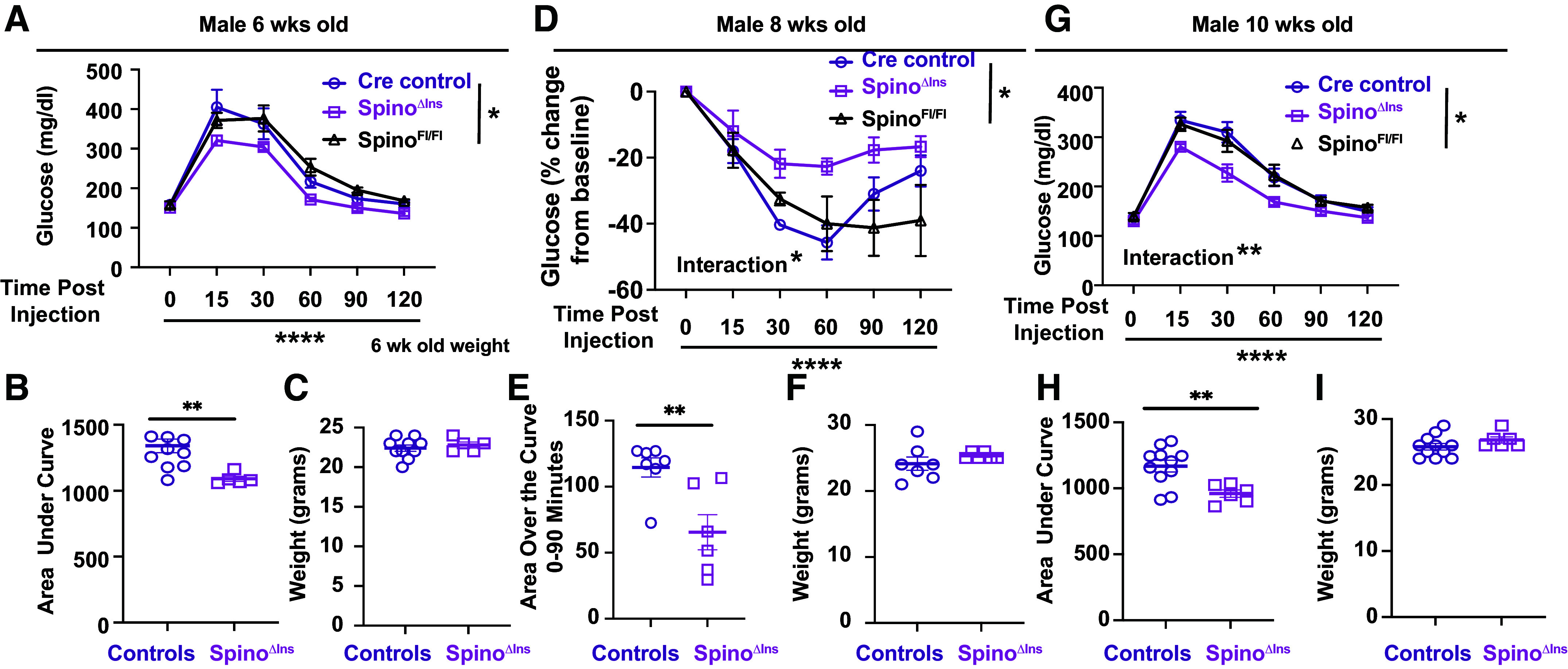
Loss of spinophilin, specifically in β cells, improves glucose tolerance and reduces insulin tolerance. *A*: glucose tolerance test (GTT) of 6-wk-old β cell-specific spinophilin knockout (KO) mice (Spino^ΔIns^) with Cre and flox controls. *B*: area under the curve for GTT of 6-wk-old Spino^ΔIns^. *C*: weights for 6-wk-old Spino^ΔIns^ and controls. *D*: insulin tolerance test (ITT) (normalized to baseline) of 8-wk-old Spino^ΔIns^ and controls. *E*: area over the curve for ITT (normalized to baseline) of 8-wk-old Spino^ΔIns^ and controls. *F*: weights for 8-wk-old Spino^ΔIns^ and controls. *G*: GTT of 10-wk-old Spino^ΔIns^ and controls. *H*: area under the curve for GTT of 10-wk-old Spino^ΔIns^. *I*: weights for 10-wk-old Spino^ΔIns^ and controls. *T* tests or two-way ANOVAs (comparing genotype, time, or a genotype × time interaction) were performed. A Grubbs’s test was performed and identified two outliers, one 10-wk-old Cre control and one 6-wk-old Spino^ΔIns^ mouse, which are not included in these data. *n* = 3–7 mice per group. *P < 0.05, **P < 0.01, ****P < 0.001. All statistics are shown in Supplemental Material (https://doi.org/10.6084/m9.figshare.22507135.v1).

Overall, the data aforementioned suggest that *Spino*^−/−^ mice are lean and have improved glucose tolerance, that obesity modulates spinophilin protein-protein interactions within the pancreas, and that spinophilin is expressed in, and interacts with, PP1, ribosomal, cytoskeletal, and enzyme proteins isolated islets. However, whether spinophilin-dependent regulation of body weight per se is the reason for the improved glucose tolerance is not clear. We crossed our recently generated and validated conditional spinophilin knockout mice (24) with a well-characterized Ins1-Cre mouse line (25) to knockout spinophilin specifically in pancreatic β cells (*Spino*^ΔIns^) to determine if loss of spinophilin specifically in pancreatic β cells in vivo impacts glucose tolerance. *Spino*^ΔIns^ mice, compared with control (*Spino*^fl/fl^ and *Ins1*^Cre^), had improved glucose tolerance at 6 and 10 wk (Fig. 7, *A*, *B*, *G*, and *H*) with no change in body weight (Fig. 7, *C*, *F*, and *I*). Suggesting loss of spinophilin specifically in pancreatic β cells improves glucose tolerance similar to *Spino*^−/−^ mice but does so without impacting body weight. There were no differences detected in the AUC between the two control groups (Supplemental File; https://doi.org/10.6084/m9.figshare.22507066.v1), so we pooled the control area under the curve and weight data. When evaluating insulin tolerance, Spino^ΔIns^ mice had no deficits in insulin tolerance tests when data were not normalized to baseline (Supplemental File). However, when normalized to baseline, Spino^ΔIns^ mice had a significantly decreased area over the curve from 0 to 90 min, demonstrating less glucose uptake upon insulin injection (Fig. 7, *D* and *E*). These data suggest loss of spinophilin specifically in pancreatic β cells may impair peripheral insulin sensitivity. It is unclear if the loss of spinophilin impacts β cell mass. To begin to address this, we measured β cell mass in the *Spino*^−/−^ mice. We found no difference in the percent β cell area within fixed pancreatic tissue (Supplemental File). This is consistent with a role for spinophilin in individual β cell function, but not overall β cell number/mass.

## DISCUSSION

Previous studies determined that *Spino*^−/−^ mice had lower body weight, increased lean mass, decreased fat mass, and improved glucose tolerance, but these changes were only tested or observed in male mice (13–15). Herein, we found that global loss of spinophilin in two mouse models of obesity, *Lepr*^db/db^ and HFF-induced obesity, decreased weight gain and improved glucose tolerance in both sexes. A previous study showed no difference in weights of HFF female mice; however, they started mice on diet at an older age (8 wk vs. 4 wk) and measured weights for 8 wk compared with our 16-wk evaluation (15). This may be important as 4-wk-old mice begin an HFF diet before sexual maturity, and this younger age may be more reflective of current obesity trends in human populations. There are known sex and hormonal differences in spinophilin expression at least in the hippocampus (37) and our data suggest that body weight differences are greater at the later time points compared with earlier time points in the female mice.

Recent studies suggested that changes in adipose tissue physiology may underlie improvements in metabolic parameters (15, 23). Specifically, Dr. Hongjun Wang and colleagues found that loss of spinophilin decreased adipose tissue weight and caused browning of white adipose tissue. Therefore, some of the metabolic improvements may be associated with this effect. To our knowledge, no one has evaluated spinophilin expression in adipose tissue and whether this effect is due to spinophilin specifically in adipose tissue or is linked to other changes.

Spinophilin function is linked to the immunological synapse and can promote antigen presentation by dendritic cells and downstream T-cell activation (38). Moreover, the immune system may modulate adipose tissue browning; however, how it does so is not fully known (39, 40). Our observations that loss of spinophilin in *Lepr*^db/db^ mice decreased body weights in male and female mice to a similar extent suggest that additional mechanisms not associated with adipose tissue dysregulation of leptin hormone may be playing a role in body weight changes. Therefore, to better detail spinophilin effects specifically in adipose tissue or immune cells, future studies using our conditional spinophilin knockout mice (24) crossed with an adipose or immune-specific Cre line would help to further determine in which cell type spinophilin is playing a role in limiting adipose tissue browning.

It is unclear if spinophilin-dependent regulation of body weight and composition is the main driver of improved whole body metabolic parameters. Previous studies observed no difference in GSIS at low or high glucose in spinophilin knockout mice; however, there was an increase in GSIS in both conditions when the M3 muscarinic receptor agonist, oxotremorine-M was added, suggesting that spinophilin specifically in islets may limit GSIS under certain pharmacological stimulations (14). Therefore, as the M3 muscarinic receptor is a Gq-coupled receptor, spinophilin may modulate calcium release from intracellular ER stores; however, future studies need to detail how specifically spinophilin is regulating M3 receptor activity.

To detail a role for spinophilin specifically in β cells, we generated a Spino^ΔIns^ mouse by crossing our validated conditional knockout mice (24) with a validated Ins1-Cre mouse line (25). Loss of spinophilin specifically in β cells had no effect on weight gain but did improve glucose tolerance in chow-fed male mice. Therefore, spinophilin within islets broadly and β cells specifically can improve metabolic parameters. Moreover, these data suggest that these improved parameters are independent of the decreased body weight and improved body composition. As we used male mice exclusively for our β cell-specific studies, future studies will need to detail if loss of spinophilin in β cells impacts GTT in female mice and if this loss can rescue GTT impairments in HFF-induced glucose intolerance.

We observed more circulating glucose in response to insulin in Spino^ΔIns^ mice, contrasting previous studies that observed improved insulin-dependent glucose uptake (14, 15, 23). However, in the previous study by Ruiz de Azua and colleagues, there was a trend toward a decreased insulin secretion in response to glucose injection, which may suggest disrupted insulin secretion within the β cells of spinophilin KO mice. However, this alteration does not appear to be due to global changes in β cell mass as we did not observe any gross differences in β cell mass between WT and *Spino*^−/−^ mice. However, if Spino^ΔIns^ mice have altered β cell mass is not known and future studies can determine if cell type-specific knockout of spinophilin has a unique effect when contrasted with global knockout. Moreover, the difference between global and cell type-specific knockout mice on insulin secretion may be due to different roles of spinophilin in islets versus peripheral tissues that take up glucose. For instance, given that β cell type-specific loss improves glucose tolerance basally, Spino^ΔIns^ mice may have greater circulating insulin that could lead to downstream adaptive changes due to basally higher amounts of secreted insulin.

To begin to understand how obesity impacts the pancreatic spinophilin protein interactome, we immunoprecipitated spinophilin from whole pancreas of control and Lepr^db/db^ mice, and performed a “GelC-MS” proteomics approach where we excised spinophilin immunoprecipitates from a Coomassie-stained gel. We detected multiple known interactors that we have previously observed in brain tissue (PP1, neurabin, and myosin protein) and putative novel (BiP and PDI) proteins. Overall, we identified proteins associated with actin cytoskeleton organization, ER protein processing, and pancreatic secretion. Using spectral counting, we found that HFF increased the interaction of multiple proteins with spinophilin, including myosin-9 and BiP, and decreased neurabin spectral counts in the *Lepr*^db/db^ mice compared with control mice. We validated the increased interaction with myosin-9 and the decreased interaction with neurabin by immunoblotting. These data suggest that obesity impacts spinophilin interactions in the pancreas.

We followed up these studies comparing spinophilin interactions from chow and HFF WT mice using ratiometrically quantitative TMT proteomics and an advanced Orbitrap Eclipse Tribrid mass spectrometer. To further probe obesity effects on spinophilin interactions, we used a global, HFF spinophilin KO mouse to subtract nonspecific interactions. Although we observed a greater total number of interacting proteins using this approach, there was overlap in the pathways that we detected when compared with the original Gel-C MS approach, including cytoskeletal proteins, translation proteins, and pancreatic secretion. Some proteins, such as BiP were not quantitatively higher in the HFF WT compared with spinophilin KO IP, suggesting this protein is a nonspecific interactor with the spinophilin antibody or beads, but it may be generally upregulated under obese conditions. This experiment recapitulated the increased association of spinophilin with myosin-9 in obese mice. Myosins tend to be “sticky” when it comes to coimmunoprecipitation and mass spectrometry (41); however, it met our specificity and fold-change requirement cut-off, suggesting that this is a specific spinophilin interactor.

In addition to myosin-9, we observed increased spinophilin protein interactions in obesity with additional proteins involved in regulating actin dynamics, including other myosin and myosin-associated proteins, F-actin capping proteins, and actin proteins. Cytoskeletal proteins, such as class II and V myosins (36), have important roles in cytoskeletal rearrangement (35) and dense core vesicle transportation (42) that alter second-phase GSIS in impaired β cells, a long-term insulin secretion that requires movement of insulin granules to the membrane for release. Cytoskeletal rearrangement is known to contribute to the second phase of insulin secretion (35) and spinophilin is known to promote F-actin bundling via its F-actin binding domain (43). Therefore, it is possible that spinophilin may have specific impacts on the second phase of insulin secretion by enhancing the movement of the reserve pool of insulin-containing dense core vesicles. These data, along with previous studies discussed earlier showing differences in static insulin secretion from islets isolated from control and *Spino*^−/−^ mice under oxotremorine-M stimulated, but not basal, conditions demonstrate a need for future perifusion studies to detail if β cell-specific loss of spinophilin can impact the second phase of GSIS release either basally or following HFF.

We found an obesity-induced increase in spinophilin interactions with proteins involved in protein digestion and absorption in the pancreas, such as trypsin and chymotrypsin. These proteins are classically associated with exocrine pancreas function; however, they also play a role in insulin processing (44). Specifically, emerging evidence suggests that pancreatic enzymes, such as amylase, can enhance insulin-independent glucose uptake into the intestine thereby limiting its levels in the blood and thereby decreasing insulin secretion (45, 46). Moreover, in whole pancreas, spinophilin was found to specifically interact with regulators of amylase, such as proteases like trypsin, carboxypeptidases, and chymotrypsins that can decrease amylase levels and/or actions (47). Our data suggest that obesity impacts spinophilin interactions with digestive enzymes that are enriched in exocrine pancreas, which can in turn modify amylase action and intestinal uptake of glucose.

We also observed several proteins involved in translation and protein metabolism, including ribosomal subunits and complement proteins. These proteins may be important in insulin processing and GPCR signaling (48). Spinophilin and GPCR signaling have been heavily investigated in the brain (20, 24, 49–54), but outside of the M3 muscarinic receptor (14), how spinophilin modulates GPCRs in the β cells or in other pancreatic cell types is unclear. We also identified multiple 40S and 60S ribosomal subunits that had an obesity-dependent increased interaction with spinophilin. Both subunits must be present for functional translation (55). However, ribosomal protein-deficient cells have impaired insulin signaling (56). An additional class of proteins that had an increased interaction with spinophilin was the perilipins, which protect the β cell from another form of stress in T2D known as lipotoxicity (57). Perlipin-2 has previously been shown to regulate insulin secretion (58); however, the function of perilipin-1 and -4 detected here are less well known.

The islets are only a small component of pancreatic tissue. Therefore, to assess if the spinophilin interactome is similar in isolated islets, we immunoprecipitated spinophilin from isolated islets of three WT mice and one *Spino*^−/−^ mouse and performed a qualitative analysis of proteins that coprecipitated with spinophilin. Spinophilin had much lower spectral counts in immunoprecipitates isolated from intact islets compared with whole pancreas. Moreover, as mass spectrometry detects the most highly abundant proteins, a caveat to our immunoprecipitation approaches is that they may miss lower-abundance protein interactions and may overrepresent higher-abundance interacting proteins. However, many of the classes of interacting proteins, such as cytoskeletal proteins (e.g., actinin-1, cofilin, and annexin-A2) and ribosomal protein subunits, were similar between isolated islets and whole pancreas as well as in our previous studies in the brain (18, 22, 24, 28, 59, 60).

In addition to cytoskeletal and ribosomal proteins, we again detected pancreatic enzymes such as trypsinogen, amylase, and lysozyme within isolated islets. The most likely explanation for this is that these highly abundant pancreatic enzymes are from exocrine cells that copurify with the isolated islets. Although less likely, amylase and spinophilin may be cosecreted and bound to β cells or amylase may be taken up by islet cells and interact with spinophilin in these cells. As coimmunoprecipitation studies do not permit localization of protein interactions, future immunohistochemistry studies will need to be performed to validate colocalization and test these alternative hypotheses. Amylase is known to decrease insulin secretion in cell lines and improve glucose tolerance in diabetic pigs (61). Therefore, irrespective of the location of interaction, spinophilin association and regulation of amylase and other exocrine enzyme activity may be an interesting future area of study.

Although spinophilin is postsynaptically enriched, Stephan Sigrist’s group (62, 63) has found a presynaptic role for spinophilin at the Drosophila neuromuscular junction in promoting neurotransmitter release. Therefore, determining if spinophilin plays a role in insulin release and if so, how, is an area ripe for future study. In addition, future studies will need to determine how HFF impacts spinophilin interactions within the islet and how the loss of spinophilin impacts islet-associated protein phosphorylation and function.

Although we find here that levels of spinophilin expression in peripheral tissues impacts metabolic parameters, further work is needed to understand the signaling mechanisms by which spinophilin improves glucose tolerance and may regulate insulin secretion. Specifically, how does loss of spinophilin specifically in pancreatic β cells impact HFF-induced alterations in whole body glucose tolerance as well as phasic GSIS. As PP1 is highly promiscuous, modulating PP1 interacting proteins, such as spinophilin, offers a potentially more targeted approach to modulate this pathway. For instance, the Food and Drug Administration-approved drug, Guanabenz, can act to modulate eukaryotic translation initiation factor 2 α signaling by modulating the GADD34-PP1 complex, demonstrating the potential for altering PP1 activity by targeting regulatory proteins of the phosphatase (64). Therefore, future studies modulating the spinophilin-PP1 complex, or other spinophilin protein interactions could be a novel therapeutic approach for improving metabolic function or restoring obesity-induced dysregulation of metabolic function.

## DATA AVAILABILITY

All raw and processed mass spectrometry data have been uploaded to MassIVE repository with Accession Nos. MSV000091159 and MSV000093860. Raw immunoblots or other data that are not in Supplemental Data will be provided upon request.

## SUPPLEMENTAL DATA

Supplemental Tables S1–S7: https://www.doi.org/10.6084/m9.figshare.22507135.

Supplemental File: https://doi.org/10.6084/m9.figshare.22507066.v1.

## GRANTS

This work was supported by the Histology Core of the Indiana Center for Musculoskeletal Health at IU School of Medicine and the Indiana Clinical Translational Sciences Institute (CTSI). The proteomics work was supported, in part, by the Indiana Clinical and Translational Sciences Institute Award No. UL1TR002529 from the National Institutes of Health, National Center for Advancing Translational Sciences, Clinical and Translational Sciences Award and the P30 IU Simon Comprehensive Cancer Center Support Grant under Award No. P30CA082709 from the National Cancer Institute. Funding for T.L.B.-A. was from NIH-1R15EY033968. Funding for the generation of Spino^Fl/Fl^ mice and additional support comes from an R21/R33 Award from the National Institute on Drug Abuse R21/R33 DA041876 (to A.J.B.), Department of Biology/School of Science at Indiana University-Purdue University Indianapolis (IUPUI) (to K.C.S. and A.J.B.), Department of Pharmacology and Toxicology Startup Funds (to A.J.B.), Strategic Research Initiative Funds Indiana University School of Medicine and Stark Neurosciences Research Institute (to A.J.B. and K.C.S.), and Center for Diabetes and Metabolic Diseases Pilot and Feasibility proposal (to A.J.B.). Funding was also received from the National Institute on Diabetes and Digestive and Kidney Diseases Grant P30DK097512.

## DISCLOSURES

No conflicts of interest, financial or otherwise, are declared by the authors.

## AUTHOR CONTRIBUTIONS

K.C.S., N.R.S., E.T.C., K.S.O., A.L.M., E.H.D., T.L.B.-A., and A.J.B. conceived and designed research; K.C.S., N.R.S., E.T.C., K.S.O., E.H.D., T.L.B.-A., and A.J.B. performed experiments; K.C.S., E.T.C., K.S.O., A.L.M., E.H.D., T.L.B.-A., and A.J.B. analyzed data; K.C.S., K.S.O., E.H.D., and A.J.B. interpreted results of experiments; K.C.S., E.H.D., and A.J.B. prepared figures; K.C.S., E.T.C., E.H.D., T.L.B.-A., and A.J.B. drafted manuscript; K.C.S., K.S.O., A.L.M., E.H.D., T.L.B.-A., and A.J.B. edited and revised manuscript; K.C.S., N.R.S., E.T.C., K.S.O., A.L.M., E.H.D., T.L.B.-A., and A.J.B. approved final version of manuscript.
